# Special Issue: Antimicrobial Activity of Plants Against Emerging or Drug-Resistant Human Pathogens

**DOI:** 10.3390/antibiotics15101001

**Published:** 2026-10-09

**Authors:** Gloria María Molina-Salinas, Andrés Humberto Uc-Cachón

**Affiliations:** Unidad de Investigación Médica Yucatán, Instituto Mexicano del Seguro Social, Mérida 97150, Yucatán, Mexico

## Closing Editorial

Plant-derived antimicrobial agents have gained increasing scientific attention as antimicrobial resistance continues to rise globally, reducing the effectiveness of conventional antibiotics and complicating the management of emerging and drug-resistant pathogens. Over the past decade, advances in phytochemical characterization, high-resolution microbiological assays, and synergistic evaluations with existing antibiotics have expanded our understanding of the mechanisms by which natural compounds exert antimicrobial effects [1,2,3,4]. These developments have highlighted the potential of plant metabolites to disrupt biofilms [3], interfere with quorum sensing, inhibit efflux pumps, and destabilize microbial membranes—mechanisms that are particularly relevant for pathogens that persist within human hosts or clinical environments.

Despite these advances, several gaps remain. Variability in metabolite composition, driven by plant genetics, environmental conditions, and extraction methods, continues to challenge the reproducibility and standardization of these analyses. Toxicological and pharmacokinetic data are often incomplete, thereby limiting translational progress. Mechanistic studies often rely on phenomenological observations rather than detailed molecular analyses, leaving uncertainties regarding how plant-derived compounds interact with microbial physiology and host immunity [5]. Addressing these gaps is essential for advancing the regulatory and clinical viability of natural antimicrobials.

The contributions to this Special Issue provide valuable insights into these challenges. Several studies have highlighted the antimicrobial activity of plant extracts and essential oils against clinically relevant pathogens, including Gram-positive and Gram-negative bacteria, multidrug-resistant urinary isolates, and antibiotic-resistant *Escherichia coli* [6,7,8,9,10]. Others have isolated and characterized novel bioactive compounds and explored the inhibition of biofilm formation using plant-derived metabolites [8,9]. Complementing these experimental studies, comprehensive reviews have examined synergistic strategies between plant compounds and conventional antibiotics, antibiofilm phytochemicals, and the broader landscape of natural antibacterial agents found in food plants [11,12].

Recent ethnopharmacological studies have also demonstrated the importance of linking traditional medicinal knowledge with mechanistic microbiological and in silico validation, an approach strengthened by recent investigations on Mayan medicinal plants, such as *Krugiodendron ferreum*, which revealed anti-staphylococcal activity and interference with resistance and virulence pathways [13].

Future research should prioritize the development of harmonized protocols for extract preparation and chemical characterization, alongside comprehensive toxicological and pharmacokinetic evaluations. In addition, omics-based approaches should be increasingly applied to elucidate mechanisms of action at the molecular level and strengthen the translational potential of plant-derived antimicrobials. Advanced *in vitro* systems and humanized models may help bridge the gap between experimental findings and their clinical applicability. Expanding collaborative networks across regions and disciplines is essential for generating robust datasets that support translational and regulatory advancements.

In conclusion, this Special Issue gathers contributions from researchers across multiple continents, reflecting the global interest in plant-derived antimicrobials as part of the broader response to antimicrobial resistance. The findings presented here highlight both the promises and challenges of this rapidly advancing field and reinforce the need for continued interdisciplinary research to develop effective, sustainable, and innovative solutions to one of the most pressing threats to human health.
